# Inequalities in Exclusively Mobile Interventions Targeting Weight‐Related Behaviors: Systematic Review of Observational Studies

**DOI:** 10.1111/obr.70057

**Published:** 2025-12-06

**Authors:** Laura M. König, Cynthia C. Forbes, Heide Busse, Ann DeSmet, Dorothy Szinay, Jin Wan, Zhirui Guo, Eline S. Smit

**Affiliations:** ^1^ Faculty of Psychology University of Vienna Vienna Austria; ^2^ Faculty of Life Sciences University of Bayreuth Kulmbach Germany; ^3^ Wolfson Palliative Care Research Centre, Hull York Medical School University of Hull Hull UK; ^4^ Leibniz Institute of Prevention Research and Epidemiology – BIPS Bremen Germany; ^5^ Leibniz ScienceCampus Digital Public Health Bremen Germany; ^6^ Faculty of Psychology and Educational Sciences Université Libre de Bruxelles Brussels Belgium; ^7^ Department of Communication Studies University of Antwerp Antwerp Belgium; ^8^ Behavioural Science and Health University College London London UK; ^9^ Amsterdam School of Communication Research/ASCoR, Department of Communication Science University of Amsterdam Amsterdam the Netherlands; ^10^ Erasmus School of Health Policy and Management, Department of Health Technology Assessment Erasmus University Rotterdam Rotterdam the Netherlands

**Keywords:** body weight, digital divide, health promotion, mHealth, social inequality

## Abstract

Mobile health interventions are promising behavior change tools, but they might benefit deprived populations less due to disparities in intervention uptake, engagement, and effectiveness. Evidence so far mainly stems from clinical trials, which may suffer from selection bias. This systematic review investigated differences in uptake of, engagement with, and effectiveness of exclusively mobile interventions for diet, physical activity, and sedentary behavior in adults and real‐life contexts. Five databases (CINAHL, EMBASE, PsycINFO, PubMed, and Web of Science) were searched from inception to November 2023. Records were independently screened by two authors. Observational studies including adults were considered if they reported on uptake, engagement, or effectiveness of an exclusively mobile intervention and examined outcomes by at least one inequality indicator included in the PROGRESS‐Plus framework. Of the 9707 identified records, 87 publications reporting on 88 studies were included. Most studies reported on intervention uptake and examined multiple inequality indicators. Younger age and higher socioeconomic position were mostly associated with increased uptake, although these differences did not translate to engagement or effectiveness. Results for other inequality indicators were mixed, and some (e.g., migration and sexual orientation) were rarely studied. Evidence regarding social inequality remains mixed, although some barriers to uptake, such as access to the required technology and digital literacy, exist. Research urgently needs to address potential inequalities beyond age, gender/sex, and socioeconomic position to ensure that mobile interventions do not widen existing health inequalities.

**Abbreviations**
BMI: Body Mass Index
OSF: Open Science Framework
PRISMA: Preferred Reporting Items of Systematic Reviews and Meta‐analysis
SEP: socioeconomic position

## 1 Introduction

Mobile health (mHealth) interventions are a specific form of digital health interventions that use mobile, often Internet‐supported, tools such as smartphone applications, tablets, and wearables to promote health and wellbeing or remotely support treatment and disease self‐management [1]. With the current widespread use of smartphones, mHealth interventions are available to a large number of people [2] and, as such, might be able to increase access to health information, support and care [3]. Weight‐related behaviors like physical activity, sedentary behavior, and diet are important precursors for physical and mental health, illness, and mortality [4, 5, 6, 7]. mHealth interventions also have certain important benefits; for example, advice or support can be received quickly at any time and place, personalization and tailoring of intervention content are usually possible, and the user can manage their health and wellbeing in an autonomous way. Therefore, it comes as no surprise that many mHealth interventions have been developed and evaluated that aim to promote sufficient physical activity and a healthy diet and to prevent low levels of sedentary behavior (e.g., [8, 9]).

To achieve an impact on population health, mHealth interventions must not only reach the target group but at the same time be accessible and easy to use. Intervention users should also show sufficient objective (e.g., uptake and continued usage as long as needed) and subjective engagement (e.g., enjoyment and perceived usefulness) with the intervention. Moreover, the interventions also need to be effective in promoting the desired behavioral change [10]. Previous research has shown that mHealth interventions can be as effective as face‐to‐face interventions in increasing physical activity [11, 12] and reducing sedentary behavior [13], yet uptake and engagement are not often reported [14]. Furthermore, when intervention uptake is discussed, the information usually relates to the included sample size only and does not relate to the extent to which the sample is representative of the target population or at least comparable to nonusers [15].

This is problematic, as evidence about a digital health divide exists, that is, the phenomenon that digital interventions tend to benefit the more affluent and privileged users more than their less‐privileged counterparts [16]. Indeed, based on an extensive systematic review, Western et al. [17] concluded that digital interventions promoting physical activity are effective among adults of high socioeconomic position (SEP) yet not among adults with low SEP. Besides these differences in intervention effectiveness, research has shown that less privileged populations, for example, people with lower levels of education, a lower income, older age, ethnically minoritized populations, and people from rural areas, show lower uptake and less continuous use of mHealth interventions (e.g., [18]). This further limits the potential public health impact of these interventions. Although digital health interventions have been previously described as having the potential to decrease existing inequalities for people with lower SEP, for example, when it concerned smoking cessation [19], these recent findings indicate that mHealth interventions might in fact contribute to widening inequalities between more and less privileged populations.

In a previous systematic review [20], our team made a first contribution to determine whether a digital health divide indeed exists by investigating whether the uptake of, engagement with, and effectiveness of mobile interventions for weight‐related behaviors (i.e., diet, physical activity, and sedentary behavior) differed depending on a predetermined set of inequality indicators (i.e., socioeconomic position, age, gender, level of education, health and digital literacy, sexual orientation, health services accessibility, or geographical location). Importantly, this previous review revealed that engagement with and effectiveness of mobile interventions may be influenced by certain sociodemographic variables. We found, for example, that older adults, adults in lower‐skilled jobs, and those living in rural areas might benefit less from mHealth interventions aimed at weight loss than their younger counterparts, adults who hold higher skilled jobs, or people who live in urban areas.

The idea that a broad range of an individual's characteristics may be associated with inequalities in healthcare is also captured in the Cochrane PROGRESS‐Plus framework [21], which is the predominant framework for investigating health inequalities in systematic reviews, also due to its broad scope [22]. PROGRESS refers to place of residence, race/ethnicity/culture/language, occupation, gender/sex, religion, education, socioeconomic status, and social capital. Plus refers to personal characteristics associated with discrimination (including age), features of relationships, and time‐dependent relationships, that is, instances where people temporarily are at a disadvantage.

As our previous systematic review [20] and other reviews (e.g., [23]) show, evidence of a digital health divide is inconclusive and needs further investigation, especially regarding factors going beyond age and gender, for example, by applying PROGRESS‐Plus. A potential explanation for the inconclusive findings may be that we only included experimental research designs, the majority of which were randomized controlled trials (RCTs). These RCTs consistently reported findings that are different from studies applying other research designs (e.g., single‐group pre–post studies). Although RCTs are considered the gold standard in evaluating intervention effectiveness and were thus the focus of this review, their samples are inherently biased regarding several indicators of social inequality including ethnicity and race, location, and age and thus typically not representative of the general population [24]. This is because enrolment, participation, and retention in RCTs may have inherent barriers such as information about the trial being difficult to understand for people with low levels of (health) literacy and burden due to time commitment [25, 26]. Furthermore, depending on how a trial is advertised, this advertisement already introduces biases that may bias conclusions especially regarding intervention uptake, on which evidence regarding a digital health divide is especially lacking [20]. Data from freely available mobile interventions, such as commercial apps, may provide a more realistic picture of intervention uptake, engagement, and effectiveness in real‐life settings and thus provide an important additional data source for testing for the existence of a digital health divide [23, 27]. Therefore, to more comprehensively answer the question of whether a digital health divide exists when it comes to mHealth interventions for weight‐related behaviors, we examined whether the uptake of, engagement with, and effectiveness of digital interventions for diet, physical activity, and sedentary behavior differed depending on potential inequality indicators, based on evidence from any quantitative observational study.

## 2 Methods

The review followed the Preferred Reporting Items of Systematic Reviews and Meta‐Analyses (PRISMA) guidelines [28]. The study protocol was registered on the International Prospective Register of Systematic Reviews (PROSPERO; CRD42021290769) prior to conducting the search. Raw extracted data, reasons for exclusion at the full‐text screening stage, and analysis scripts can be downloaded from the project's Open Science Framework page (OSF; https://osf.io/qs5vy/).

### 2.1 Eligibility Criteria

To be included in this systematic review, studies had to focus exclusively on adults aged 18 and over with no apparent preexisting medical condition. Studies furthermore had to report on the uptake of, engagement with, or effectiveness of exclusively mobile interventions (e.g., smartphone apps, wearables, or mobile games without any intervention components that were delivered through other means) for the promotion of weight‐related behaviors (physical activity, sedentary behavior, and diet) or weight loss. Results had to be reported separately by at least one of the following inequality indicators taken from the PROGRESS‐Plus criteria [21]: place of residence, race/ethnicity/culture/language, occupation, gender/sex, religion, education, socioeconomic position (e.g., income), social capital (e.g., relationships), personal characteristics associated with discrimination (e.g., age and disability), features of relationships (e.g., excluded from school), or other time‐depending relationships where an individual may be temporarily at a disadvantage. Only studies published in English were eligible. Studies were excluded if they reported interventions that included intervention components using any other means of delivery (e.g., face‐to‐face contact) or focused on patient populations, children, or adolescents. Systematic reviews, meta‐analyses, and study protocols were excluded, as were conference abstracts and records for which no full text could be retrieved.

### 2.2 Search Strategy

The electronic searches were conducted by CF in consultation with an information specialist from the University of Hull. The following databases were searched: PsycINFO (Ovid), MEDLINE (Ovid), EMBASE (Ovid), CINAHL (EBSCO), and Web of Science Core Collection (see the Supporting Information for the full MEDLINE search strategy). MeSH terms were developed to search for all key concepts and modified for each database. Keyword searches restricted to abstract, title, and keyword headings were also completed. Boolean logic was used to combine the terms. The search strategy can be found on the project's OSF page. Databases were initially searched from inception to 29 November 2021 with no language or country limits applied. The search was updated on 29 November 2023 to include studies from 29 November 2021 onwards. Additionally, we conducted a backward and forward citation search of all initially included studies through an electronic search on Google Scholar.

### 2.3 Screening

All records identified by the electronic search were exported to Endnote 20, deduplicated, and uploaded into Covidence (Covidence Systematic Review Software, Veritas Health Innovation, Melbourne, Australia). Titles and abstracts of all studies were screened independently by two reviewers each (L.K., C.F., J.W., and Z.G.). Afterwards, full texts were screened by two reviewers (L.K., C.F., J.W., and Z.G.). Disagreements were resolved by discussion.

### 2.4 Data Extraction and Synthesis

A data extraction form was developed based on guidelines from the Cochrane Collaboration [29]. Study characteristics (first author, paper title, year of publication, journal, study aim, study design, geographical setting of the study, and inclusion and exclusion criteria), information about setting and population (including sampling strategy and recruitment), target behaviors and social inequality indicators, the intervention (modes of delivery and description of intervention content), the sample (sample size and sociodemographic and health‐related characteristics), operationalization of outcomes, and the main findings related to the research questions for this systematic review were extracted. Data extraction was performed independently by two reviewers. Disagreements were resolved by discussion. Narrative synthesis was conducted to summarize the findings of the studies for the identified inequality indicators by outcome (uptake, engagement, and effectiveness).

### 2.5 Quality Appraisal

The quality of included studies was assessed for risk of bias using the National Institutes of Health (NIH) tool for observational cohort and cross‐sectional studies [30]. This tool is composed of 14 questions focusing on concepts that are key to a study's internal validity. For each question, reviewers provide a rating, with the option to indicate that the question is not applicable (NA), that the answer cannot be determined based on the information provided in the paper (CD), or that the issue addressed by the question is not reported on at all (NR). For the specific purpose of the present review, instead of providing a rating of *good*, *fair*, or *poor* for each question, we answered each question with *yes* or *no* as the middle option was experienced as very prone to subjectivity by reviewers. Also, we decided to split four of the 14 questions into subquestions (i.e., 4a,b; 5a,b; 9a–c; and 11a–c) as this allowed us to provide a more specific answer to these questions and thus provide a more accurate risk of bias assessment.

Because the tool does not provide explicit guidelines for assigning an overall risk of bias rating to a study but rather advises that each study should be assessed individually based on the details reported [30], after the scoring of the papers, we proceeded with a color‐coded approach. Specifically, we categorized the answers into three levels: green (i.e., when answered with *yes* or with *NA* given the study design), yellow (i.e., when not all subquestions were answered as *yes*), and red (i.e., for *no* answers). We then counted the number of greens per study to provide an overall quality rating (i.e., *poor* for 6 greens or less; *fair* for 7–10 greens; and *good* for 11–14 greens).

The risk of bias assessments of the studies was conducted independently by two reviewers per study (D.S. assessed all; a.d. and E.S. each coded half of the included studies).

## 3 Results

### 3.1 Study Selection

**FIGURE 1:**
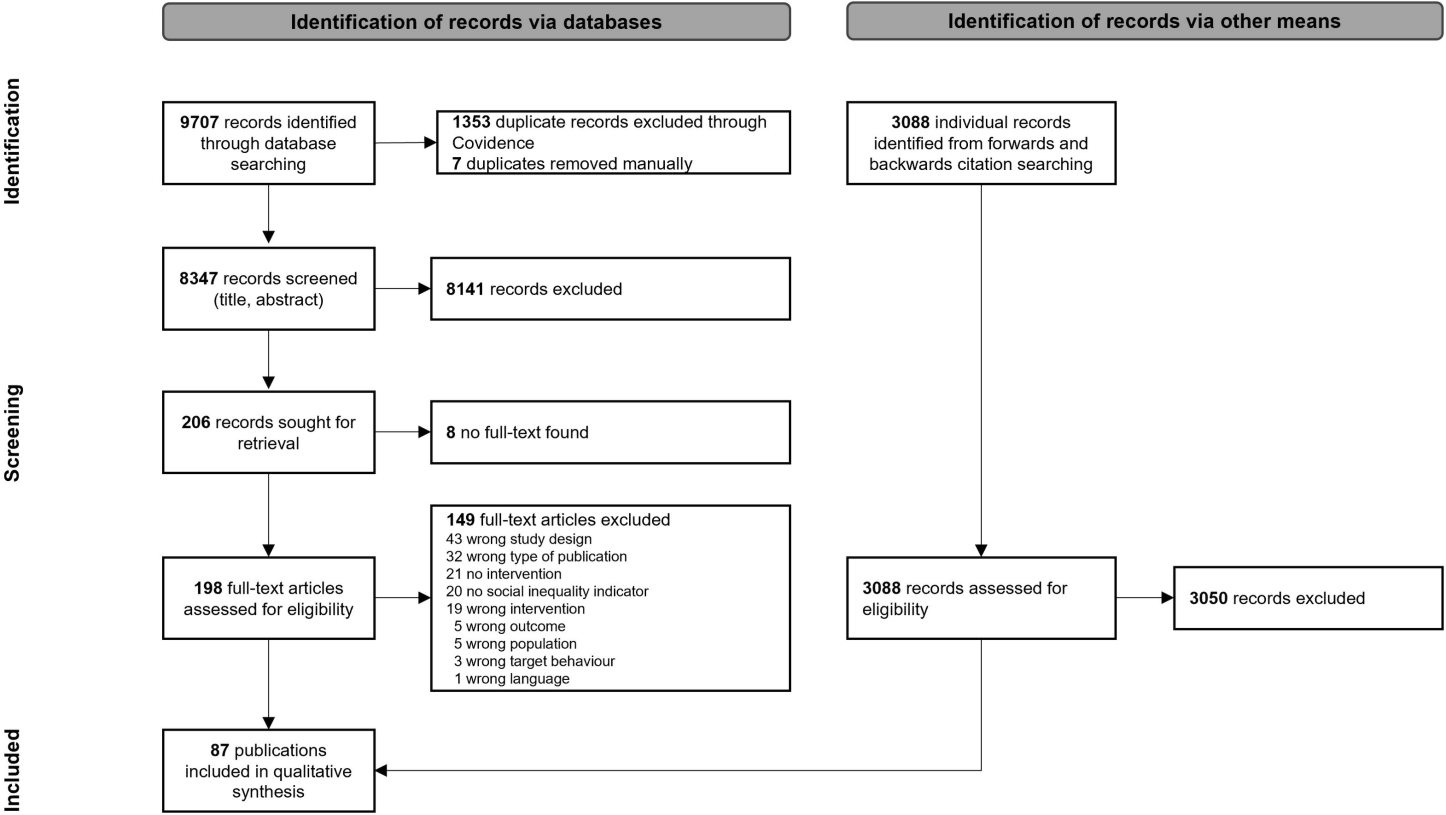
PRISMA flowchart indicating the number of records at each step of the screening process and reasons for exclusion at the full text screening stage.

Including the update, a total of 9707 records were retrieved after removing duplicates. From these, 203 were included in the full text screening, and 49 publications were included in the review. Through handsearching, another 38 publications were identified, yielding a total of 87 publications included in the narrative synthesis (see Figure 1). Because one publication reported on two separate datasets, the final number of studies included in the narrative synthesis is *K* = 88. A list of studies excluded at the full‐text screening stage and the reason for exclusion can be downloaded from the OSF (https://osf.io/qs5vy/).

### 3.2 Study and Intervention Characteristics

Studies were mostly conducted in the United States (*k* = 31 only in the United States [31, 32, 33, 34, 35, 36, 37, 38, 39, 40, 41, 42, 43, 44, 45, 46, 47, 48, 49, 50, 51, 52, 53, 54, 55, 56, 57, 58, 59, 60, 61]; *k* = 2 the United States and Canada [62, 63]; *k* = 1 the United States and South Korea [64]). Others used international samples without specifying the countries of origin [65, 66, 67, 68, 69, 70, 71, 72, 73, 74, 75, 76] (*k* = 12) or samples from Germany [77, 78, 79, 80], the Netherlands [81, 82, 83, 84], the United Kingdom [85, 86, 87] (each *k* = 4), Canada [88, 89, 90], China [91, 92, 93], Japan [94, 95, 96], Saudi Arabia [97, 98, 99] (each *k* = 3), Australia [100, 101], Poland [102, 103], Thailand [104, 105] (each *k* = 2), Brazil [106], Ethiopia [107], Hong Kong [108], Malaysia [109], Singapore [110], Sweden [111], and Taiwan [112] (each *k* = 1). Five studies did not specify where data were collected [113, 114, 115, 116, 117]. Sample sizes ranged between *N* = 29 and *N* = 12363555, with a median of *N* = 1368 participants.

Most included studies were cross‐sectional surveys [33, 39, 40, 44, 45, 46, 47, 50, 53, 54, 58, 60, 64, 70, 77, 81, 82, 83, 84, 87, 88, 93, 97, 98, 99, 101, 102, 103, 106, 107, 109, 111, 117], reported on single waves of cohort studies or longitudinal surveys [34, 35, 36, 37, 42, 43, 52, 56, 61, 79, 85, 89, 90, 94, 96, 100, 108], a survey embedded in a clinical trial [41], or surveys among users of a healthcare system or provider [38, 51, 55, 57, 59, 95, 104, 105, 110, 112]. Finally, some studies used data from commercial mobile apps such as MyFitnessPal [31, 80], Noom [32, 48, 49, 66, 67, 68, 69, 71, 74], LoseIt [62, 65, 72, 73], and others [63, 75, 76, 78, 86, 91, 92, 113, 114, 115, 116].

Most studies investigated smartphone apps only [31, 32, 33, 34, 35, 38, 41, 42, 43, 45, 47, 48, 49, 53, 56, 57, 59, 60, 62, 63, 65, 66, 67, 68, 69, 70, 71, 72, 73, 74, 75, 76, 77, 78, 79, 80, 81, 82, 83, 85, 86, 91, 92, 93, 94, 95, 96, 97, 98, 99, 100, 102, 104, 105, 106, 107, 108, 109, 112, 114, 115, 117], whereas some investigated wearables only (including smartwatches) [36, 37, 40, 44, 46, 50, 51, 54, 55, 58, 61, 64, 88, 90, 101] or both [39, 52, 84, 87, 89, 103, 110, 111, 113]. Three studies also included smart scales [103, 113, 116].

Thirty‐eight studies focused on diet as the target behavior [31, 33, 34, 35, 38, 39, 42, 43, 45, 52, 53, 56, 59, 60, 62, 63, 65, 67, 72, 75, 77, 79, 80, 81, 89, 97, 99, 101, 102, 103, 104, 107, 108, 109, 112, 114, 115, 117], 57 studies focused on physical activity [33, 34, 35, 36, 37, 39, 42, 43, 44, 45, 46, 47, 50, 51, 52, 53, 54, 55, 56, 58, 59, 62, 63, 64, 67, 70, 72, 76, 77, 78, 79, 81, 82, 83, 84, 85, 86, 87, 88, 89, 90, 91, 92, 94, 95, 96, 97, 98, 99, 101, 103, 104, 107, 108, 109, 110, 111, 118], and 36 studied weight [32, 39, 41, 42, 45, 48, 49, 52, 53, 56, 57, 61, 63, 65, 66, 67, 68, 69, 71, 72, 73, 74, 75, 77, 93, 97, 98, 99, 103, 105, 106, 108, 113, 114, 116, 117]. The majority of studies focused on one target behavior, but 18 studies addressed two [33, 34, 35, 43, 59, 62, 65, 75, 79, 81, 89, 90, 98, 101, 104, 107, 109, 114, 117], and 14 addressed all three target behaviors simultaneously [39, 42, 45, 52, 53, 56, 63, 67, 72, 77, 97, 99, 103, 108].

The majority of studies focused on mobile intervention uptake (*k* = 53) [33, 34, 35, 36, 37, 38, 39, 42, 43, 44, 45, 47, 49, 52, 53, 54, 55, 56, 58, 59, 61, 62, 64, 70, 74, 77, 79, 81, 82, 83, 84, 85, 86, 87, 88, 89, 93, 96, 97, 98, 99, 100, 102, 103, 106, 107, 108, 109, 111, 116, 117], followed by engagement (*k* = 31) [38, 40, 46, 50, 51, 52, 63, 65, 66, 67, 70, 72, 73, 74, 75, 76, 78, 80, 83, 85, 86, 88, 90, 92, 93, 97, 100, 101, 104, 110, 114, 115] and effectiveness (*k* = 29) [31, 32, 41, 46, 48, 57, 60, 62, 63, 66, 67, 68, 69, 71, 75, 76, 85, 86, 91, 94, 95, 99, 100, 105, 112, 113, 114, 115, 116], with 22 studies investigating several of these endpoints [38, 46, 52, 62, 63, 66, 67, 70, 74, 76, 82, 85, 86, 88, 93, 97, 99, 100, 114, 115, 116, 119]. Most studies included gender or sex (*k* = 79), age (*k* = 74) and various indicators of SEP including education (*k* = 41), income (*k* = 26), employment or occupation (*k* = 17), or SEP composite scores (*k* = 7). A total of 25 studies reported on potential racial or ethnic disparities. Thirteen studies included location; most either compared urban and rural areas within a country or compared countries or continents. Migration status was taken into account in *k* = 4 studies. Some studies also included a range of relationship factors such as marital or relationship status (*k* = 17), having children (*k* = 6), cohabiting (*k* = 4), having sick family members (*k* = 1), or sexual orientation (*k* = 1). Finally, a small number of studies also addressed health‐related social inequality indicators such as having chronic conditions or comorbidities (*k* = 14), health insurance coverage or utilization (*k* = 7), regular provider or primary care visits (*k* = 3), and disability status (*k* = 1). See Table 1 for study characteristics and Table 2 for a breakdown of social inequality indicators by outcome.

**TABLE 1:** Overview of included studies.

First author, year	Target behavior(s)	Inequality indicators	Study design	Geographical setting	Mode of delivery	Number of participants
Akerberg, 2017 [111]	Physical activity	Gender/sex, age, health status, disability, education	Cross‐sectional survey	Sweden	Physical activity self‐monitoring technologies	107
Alshathri, 2020 [97]	Diet, physical activity, weight	Age, gender/sex, region, education, employment, income, health status	Cross‐sectional web‐based survey	Saudi Arabia	Smartphone app	1074
Althoff, 2022 [31]	Diet	Location, income, education	Cross‐sectional study	United States	Smartphone app	1,164,926
Amer, 2022 [98]	Weight, physical activity	Gender/sex, marital status, having children, age, education, nationality, occupation, chronic conditions	Cross‐sectional study (survey)	Saudi Arabia	Smartphone app	679
Behr, 2023 [32]	Weight	Age	Longitudinal study with cross‐sectional survey	United States	Smartphone app	7495
Ben Neriah, 2019 [65]	Diet, weight	Age, gender/sex	Cross‐sectional analysis of baseline data	International	Smartphone app	175,402
Bender, 2014 [33]	Diet, physical activity	Ethnicity/race, age, education, relationship factors, gender/sex, marital status	Cross‐sectional survey	United States	Smartphone app	904
Bhuyan, 2016 [34]	Physical activity, diet	Age, gender/sex, income, health status, marital status, education, occupation, health services accessibility, location	Secondary analysis of date in Health Information National Trends Survey (HINTS)	United States	Smartphone apps	3677
Bol, 2018 [81]	Diet, physical activity	Age, education, gender/sex	Cross‐sectional survey	The Netherlands	Smartphone app	1079
Broom, 2018 [85]	Physical activity	Age, gender/sex	Survey with repeated measures design	United Kingdom	Smartphone app	461
Carroll, 2017 [35]	Physical activity, diet	Age, gender/sex, ethnicity/race, income, level of education, English proficiency, BMI	Secondary analysis of HINTS data	United States	Smartphone app	3584
Chandrasekaran, 2020 [36]	Physical activity	Age, gender/sex, education, ethnicity/race, marital status, income, frequency of provider visits, presence of chronic conditions	Longitudinal cohort	United States	Wearable device	4551
Chandrasekaran, 2021 [37]	Physical activity	Gender/sex, education, ethnicity/race, marital status, income, number of chronic conditions, frequency of provider visits	Longitudinal cohort	United States	Wearable device	1481
Cheng, 2022 [112]	Diet	Age, sex, marital status, education	Retrospective cohort study	Taiwan	Smartphone app	45
Chin, 2016 [66]	Weight	Age, gender/sex	Retrospective cohort study	International	Smartphone app	35,921
Coughlin, 2022 [38]	Diet	Gender/sex, race, education, income, having children, age	Secondary analysis from a 6‐month, multisite, cohort study	United States	Smartphone app	1017
Dallinga, 2015 [82]	Physical activity	Gender/sex, age	Cross‐sectional study	The Netherlands	Smartphone app	3800.
De Bruijn, 2021 [83]	Physical activity	Age, gender/sex, education	Cross sectional survey	The Netherlands	Smartphone app	1683
Drake, 2020 [39]	Diet, physical activity, weight	Age, gender/sex, health status, income, education, health services accessibility, social capital, ethnicity/race	Survey	United States	Smartphone apps, wearables	1329
Ernsting, 2017 [77]	Physical activity, diet, weight	Gender/sex, age, occupation, education, income, first language	Cross‐sectional survey	Germany	Smartphone app	4144
Fischer, 2022 [78]	Physical activity	Age, gender/sex, marital status, education, employment	Single‐arm observational study	Germany	Smartphone app	97
Friel, 2020 [40]	Physical activity	Age, gender/sex, race, education, income, cohabitation, employment	Cross‐sectional survey	United States	Wearable activity trackers	2002
Goldstein, 2021 [41]	Weight	Age, gender/sex, ethnicity	Secondary analysis of trial data	United States	Smartphone app	116
Gorny, 2022 [110]	Physical activity	Ethnicity, education	Mixed methods study	Singapore	Smartphone app and activity tracker	29
Hahn, 2022 [42]	Diet, physical activity, weight	Gender/sex, SEP, ethnicity/race, age, education	One time point of longitudinal population‐based study	United States	Smartphone app	1428
Hahn, 2022 [43]	Diet, physical activity	Gender/sex	One time point of longitudinal population‐based study	United States	Smartphone app	1446
Hamaya, 2021 [94]	Physical activity	Age, gender/sex	Longitudinal cohort	Japan	Smartphone app	12,602
Hamaya, 2022 [95]	Physical activity	Gender/sex	Analysis of available health data	Japan	Smartphone app	15,662
Han, 2021 [68]	Weight	Age, gender/sex	Retrospective cohort study	International	Smartphone app	23,682
Han, 2021 [67]	Diet, physical activity, weight	Income of countries, gender/sex, age	Retrospective cohort study	International	Smartphone app	8343
Hendrie, 2020 [100]	Diet	Age, gender/sex	Cohort study	Australia	Smartphone app	5092
Hill, 2018 [62]	Diet, physical activity	Gender/sex, age	Repeated measures cross sectional analyses	United States, Canada	Smartphone app	7007
Huang, 2022 [91]	Physical activity	Age, gender/sex, location, health status	Cross sectional survey	China	Smartphone app	643
Inchusri, 2023 [104]	Diet, physical activity	Sex, generation (age group)	Secondary analysis of data from prospective cohort study	Thailand	Smartphone app	827
Jabour, 2021 [99]	Physical activity, diet, weight	Gender/sex	Cross‐sectional survey	Saudi Arabia	Smartphone app	383
Jacobs, 2017 [69]	Weight	Age, gender/sex, geographical location	Longitudinal cohort	International	Smartphone app	7680
Janssen, 2017 [84]	Physical activity	Age, gender/sex, education	Cross‐sectional survey	The Netherlands	Smartphone apps, wearables	2172
Jiang, 2022 [92]	Physical activity	Age, gender/sex, education, income	Cross sectional survey	China	Smartphone app	632
Kinney, 2019 [44]	Physical activity	Gender/sex, ethnicity/race	Cross‐sectional survey	United States	Wearables	356
König, 2018 [79]	Physical activity, diet	Age, gender/sex, education	Cross‐sectional analysis of wave 4 of longitudinal cohort study	Germany	Smartphone app	1054
Krebs, 2015 [45]	Physical activity, diet, weight	Age, ethnicity/race, income, education, gender/sex, chronic diseases	Cross‐sectional survey	United States	Smartphone app	1604
Labonté, 2022 [63]	Diet, physical activity, weight	Gender/sex, age	Repeated measures cross sectional analysis	United States, Canada	Smartphone app	9372
Lee, 2020 [109]	Diet, physical activity	Age, gender/sex, education, marital status, chronic conditions, living status	Cross‐sectional survey	Malaysia	Smartphone app	4504
Lewis, 2020 [46]	Physical activity	Gender/sex, age, ethnicity/race, chronic health condition	Observational survey embeeded into mixed method study	United States	Wearables	47
Liao, 2022 [47]	Physical activity	Employment, education, income, age, marital status, insurance status, chronic disease conditions	Cross‐sectional online survey	United States	Wearable	497
Litman, 2015 [70]	Physical activity	Age, education, income	Cross sectional survey	International	Smartphone app	726
Macridis, 2018 [88]	Physical activity	Age, gender/sex, region, education, marital status	Cross‐sectional survey via telephone interview	Canada	Wearables	1215
Maher, 2017 [101]	Physical activity, diet	Age, gender/sex, education, relationship status	Cross‐sectional survey	Australia	Wearables	237
May, 2023 [48]	Weight	Gender/sex, income, marital status, having children, employment, age	Cross‐sectional study	United States	Smartphone app	840
Mitchell, 2021 [71]	Weight	Number of children, gender/sex	Longitudinal cohort	International	Smartphone app	2225
Mitchell, 2023 [49]	Weight	Age, gender/sex, region, insurance type	Retrospective longitudinal cohort study	United States	Smartphone app	67,634
Modrzejewska, 2022 [102]	Diet	Age, ethnicity/race, sexual orientation	Cross sectional study	Poland	Smartphone app	1447
Molina, 2020 [76]	Physical activity	Gender/sex	Content analysis of user profiles	International	Smartphone app	682
Nuss, 2021 [50]	Physical activity	Age, gender/sex, race/ethnicity, income, education	Survey	United States	Activity trackers	288
Oba, 2023 [96]	Physical activity	Age, gender/sex, marital status, children, education, employment, income	One time point from three‐wave survey	Japan	Smartphone app	20,573
O'Loughlin, 2022 [90]	Physical activity	Age, gender/sex, SEP, Canada‐born	Longitudinal cohort	Canada	Wearables	646
O'Loughlin, 2023 [89]	Diet, physical activity	Age, education, income, employment, gender/sex	Longitudinal cohort	Canada	Smartphone app, wearables	676
Pevnick, 2016 [51]	Physical activity	Age, gender/sex/sex, ethnicity/race, occupation, comorbidities, health insurance, income, language	Cross‐sectional cohort	United States	Wearables	66,105
Pontin, 2021 [86]	Physical activity	Age, gender/sex, occupation, SEP	Secondary analysis of data in Bounts app	United Kingdom	Smartphone app	30,804
Pourzanjani, 2016 [113]	Weight	Gender/sex	Longitudinal cohort	NA	Samartphone app, wearables, smart scales	Weight *N* = 14,411, food *N* = 7369, workout *N* = 1749
Rha, 2022 [64]	Physical activity	Age, gender/sex, income, marital status, having children, location	Cross‐sectional survey	United States, Korea	Wearables	4098
Rising, 2020 [52]	Diet, physical activity, weight	Gender/sex, age, race/ethnicity, education, income, geographical location, chronic conditions	Longitudinal cohort	United States	Smartphone apps, wearables	6789
Sae‐Lee, 2023 [105]	Weight	Age, gender/sex	Secondary analysis of data from prospective cohort study	Thailand	Smartphone app	376
Sarcona, 2017 [53]	Diet, physical activity, weight	Age, gender/sex, race/ethnicity	Cross‐sectional survey	United States	Smartphone app	401
Schuster	Physical activity	Age, gender/sex, education, income, race/ethnicity	Cross‐sectional survey	United States	Wearables	1252
Serrano, 2016 [73]	Weight	Gender/sex, age	Cross‐sectional analysis	International	Smartphone app	*n*1 = 324,649 *n*2 = 324,063 *n*3 = 323,975
Serrano, 2017 [72]	Diet, physical activity, weight loss	Gender/sex, age	Cross‐sectional analysis	International	Smartphone app	*n*1 = 336,968 *n*2 = 337,262 *n*3 = 336,778
Shandhi, 2024 [55]	Physical activity	Age, gender/sex, race/ethnicity, education, employment	Cross‐sectional survey	United States	Wearables	1368
Shaw, 2024 [56]	Diet, physical activity, weight	Age, health insurance, gender/, marital status, education, income, race/ethnicity, employment status, census region	Longitudinal cohort	United States	Smartphone app	923
Shen, 2017 [108]	Physical activity, diet, weight loss	Gender/sex, age, education, income	Cross sectional survey	Hong Kong	Smartphone app	5080
Silberman, 2020 [57]	Weight	Age, gender/sex	Retrospective secondary analyses	United States	Smartphone app	683
Stehr, 2020 [80]	Diet	Age, education, income, employment, gender/sex	Cross‐sectional survey	Germany	Smartphone app	761
Strain, 2019 [87]	Physical activity	Age, gender/sex, SEP, disability, location	Cross sectional survey secondary analyses	United Kingdom	Smartphone app, wearable/smartwatch	OTT: 3688 Health Survey for England: 4539
Teferi, 2023 [107]	Diet, physical activity	Age	Cross‐sectional study	Ethiopia	Smartphone app	405
Toro‐Ramos, 2021 [74]	Weight	Age	Retrospective cohort	International	Smartphone app	130
Torres, 2022 [75]	Diet, weight	Age, gender/sex, race/ethnicity	Longitudinal cohort	International	Smartphone app	792,692
Tricas‐Vidal, 2022 [58]	Physical activity	Gender/sex, age	Nonexperimental analytical cross‐sectional study	USA	wearables	892
Valinskas, 2022 [115]	Diet	Gender/sex	Retrospective analysis of user data	United States	Smartphone app	22,022
Valinskas, 2022 [114]	Diet, weight	Gender/sex	Retrospective study	NA	Smartphone app	10,269
Valinskas, 2023 [116]	Weight	Gender/sex	Retrospective review of medical records	NA	Smart scales, smartphone app	665
Vangeepuram, 2018 [59]	Physical activity, diet	Age, gender/sex, race/ethnicity, income, insurance, education	Cross sectional survey	United States	Smartphone app	103
Vieira, 2022 [106]	Physical activity	Age, gender/sex, race, education, SEP	Cross‐sectional observational cohort	Brazil	Smartphone app	354
Wang, 2019 [93]	Physical activity	Age, gender/sex	Cross sectional survey	China	Smartphone app	1245
West, 2017 [60]	Diet	Gender/sex, age, income, education	Cross sectional survey	United States	Smartphone app	217
Xie, 2018 [117]	Diet, physical activity	Age, gender/sex, residency, income, education, and employment	Cross‐sectional survey	NA	Smartphone app	633
Xie, 2020 [61]	Physical activity	Age, gender/sex, race/ethnicity, education, marital status, income, insurance coverage, census region, metro area, number of chronic conditions	Cross sectional study	United States	Wearables	4219
Zarnowski, 2022 [103]	Diet, physical activity, weight	Gender/sex, age, education, marital status, having children, number of household members, place of residence, employment, chronic disease	Cross‐sectional survey	Poland	Smartphone apps, wearables	1070

**TABLE 2:** Summary of results per outcome. Number of studies that reported (some) statistically significant differences in the outcome based on the inequality indicator versus the total number of studies that investigated the inequality indicator with regards to the outcome, with the proportion of statistically significant findings in brackets. Studies that did not statistically test for differences were treated as nonsignificant.

Inequality indicator	Uptake (*k* = 53)	Engagement (*k* = 31)	Effectiveness (*k* = 29)
General demographic characteristics			
Gender and sex	24/47 (51%)	14/26 (54%)	14/27 (52%)
Age	32/48 (67%)	14/25 (56%)	10/18 (56%)
Race/ethnicity	9/18 (50%)	4/8 (50%)	3/3 (100%)
Migration status	2/2 (100%)	1/2 (50%)	
Location	6/11 (55%)	0/3 (0%)	3/3 (100%)
Sexual orientation	0/1 (0%)		
Socioeconomic position			
Education	23/31 (74%)	4/12 (33%)	2/4 (50%)
Income	15/18 (83%)	7/9 (78%)	2/4 (50%)
Employment or occupation	7/13 (54%)	4/6 (67%)	0/1 (0%)
SEP composite score	5/5 (100%)	0/1 (0%)	1/1 (100%)
Relationship factors			
Marital/relationship status	4/14 (29%)	1/3 (33%)	0/2 (0%)
Having children	4/5 (80%)	1/1 (100%)	0/1 (0%)
Cohabitation	2/2 (100%)	1/1 (100%)	1/1 (100%)
Having sick family members	1/1 (100%)		
Health‐related social inequality indicators			
Having chronic conditions or comorbidities	7/12 (58%)	2/3 (67%)	1/1 (100%)
Health insurance coverage or utilization	2/6 (33%)	0/1 (0%)	
Regular provider or primary care visits	0/4 (0%)		
Disability status	1/1 (100%)		

*Note:* Empty cells indicate that the inequality indicator was not studied in relation to the outcome.

### 3.3 Quality Appraisal

Out of the 87 papers, only one [51] was assessed as having a poor quality. The majority, that is, 66 papers [32, 33, 34, 35, 36, 37, 39, 40, 41, 42, 43, 44, 45, 46, 47, 50, 52, 53, 54, 55, 56, 57, 58, 60, 62, 63, 64, 65, 66, 67, 68, 69, 70, 72, 73, 74, 75, 76, 77, 78, 80, 82, 83, 84, 85, 86, 88, 89, 91, 92, 94, 97, 98, 99, 101, 103, 108, 109, 110, 111, 112, 113, 114, 115, 116, 117], were rated as of fair quality, and a total of 20 papers [31, 38, 48, 49, 59, 61, 71, 79, 81, 87, 90, 93, 95, 96, 100, 102, 104, 105, 106, 107] were rated as of good quality. An aspect that was very often rated as red was whether key potential confounding variables were measured and adjusted statistically for their impact on the relationship between exposure(s) and outcome(s). Also, although variance and effect size estimates were often provided, a power calculation was very often missing, which resulted in this aspect often being rated as yellow. Similarly, although the exposure and outcome measures were often clearly defined and implemented consistently across all study participants, it was often unclear whether valid and reliable measures were used, yielding this aspect yellow too. Aspects that were often labeled as green related to the formulation of the research question or objective and the specification and definition of the target population. Full details of the quality appraisal can be found in the Supporting Information.

### 3.4 Uptake

**FIGURE 2:**
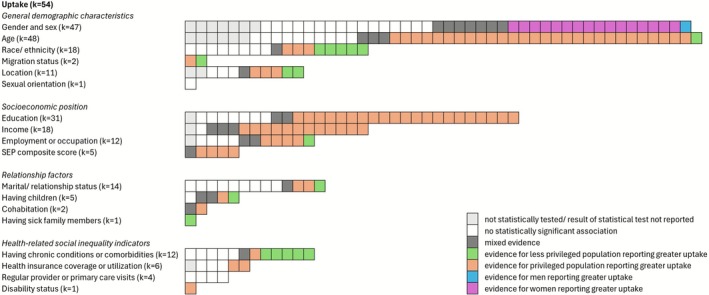
Visual summary of the included studies on intervention uptake (*k* = 54) by social inequality indicator. In each row, a box represents one study examining the relationship between the inequality indicator and intervention uptake. Privileged populations are those typically experiencing better access to healthcare and better health outcomes, such as White individuals, individuals with higher SEP as indicated by higher level of education or income or full‐time employment, individuals in a relationship but without care responsibilities, or individuals with access to health insurance and no chronic conditions. Results for genders are mixed in this regard (e.g., women reporting greater uptake of interventions but fewer benefits), and findings are thus reported separately for men and women.

A visual summary of the findings is presented in Figure 2.

#### 3.4.1 Gender and Sex

Forty‐seven studies tested for gender or sex differences in mobile intervention uptake, with 17 reporting significant differences. Almost all of these studies reported that uptake was higher in women than in men [36, 37, 39, 42, 43, 44, 52, 54, 55, 58, 61, 82, 88, 93, 109, 117]. Only Oba et al. [96] reported higher uptake rates for physical activity‐related apps in men than in women in Japan. Seven reported mixed results, typically with gender differences emerging for the uptake of some but not all mobile interventions [56, 64, 79, 84, 89, 108, 116]. For example, König et al. [79] reported gender differences in the uptake of diet but not physical activity apps. Shen et al. [108] reported opposite findings, with women having lower uptake rates only for physical activity‐related apps. In addition, Janssen et al. [84] reported higher uptake rates in women than in men for apps but not for sports watches. Rha et al. [64] suggested an interaction between gender and culture, with uptake rates being higher in women than in men in the United States but not in South Korea. Shaw et al. [56] reported gender to be associated with uptake, but this association was rendered nonsignificant if other predictors such as age, education, and health insurance coverage were included in the model. Another seven studies only reported results narratively but did not provide the results of statistical tests; all but one [72] reported higher uptake in women than in men [49, 62, 86, 97, 99, 100]. The remaining 16 studies found no gender differences in mobile intervention uptake [33, 34, 35, 38, 45, 53, 59, 77, 81, 83, 87, 98, 103, 106, 111].

#### 3.4.2 Age

Forty‐eight studies tested for age differences in mobile intervention uptake. Twenty nine reported significant differences. Almost all of these studies reported higher uptake in younger than older age groups [33, 34, 35, 36, 37, 38, 45, 52, 54, 55, 56, 61, 70, 77, 79, 81, 82, 83, 84, 85, 87, 88, 96, 98, 102, 106, 108, 109]. Only Teferi et al. [107] reported students older than 25 years to be more likely to use mobile interventions than students aged 25 years and younger. Three studies reported mixed findings. In Zarnowski et al. [103], app and wearable users tended to be younger, whereas there were no age differences for the uptake of smart scales. Hahn et al. [42] only found age differences in dietary self‐monitoring app uptake for men but not women; uptake was lower in younger versus older men. In Rha et al. [64], older age was associated with lower uptake in the United States but not the South Korean sample. Six studies summarized their findings narratively without statistically testing relationships [49, 62, 86, 97, 100]; this mainly focused on reporting mean ages or typical age ranges that do not allow drawing conclusions about potential age differences in uptake. In Alshathri et al. [97], app users were older than nonusers, whereas in Mitchell et al. [49], nonusers were somewhat older than users. Mean ages of engagement groups reported in Serrano et al. [72] all ranged between 29.7 and 30.8 years, suggesting no age differences. The remaining 10 studies reported no relationships of uptake with age [39, 47, 53, 58, 59, 74, 89, 93, 111, 117].

#### 3.4.3 Race and Ethnicity

Eighteen studies investigated relationships between race or ethnicity and mobile intervention uptake. Eight studies reported significant findings, but the direction of results was mixed. White individuals were less likely to use mobile interventions than individuals of other ethnicities in five studies [34, 35, 37, 45, 56]. Three studies, on the other hand, reported Caucasians or White individuals to be more likely to start using mobile interventions [33, 36, 38]. One study reported mixed results regarding statistical significance: For women, ethnicity was not related to intervention uptake. For men, however, Hispanic and Latino men were most likely to use weight‐related self‐monitoring apps, whereas mixed‐race men were least likely [42]. The remaining nine studies reported no significant relationships [44, 52, 53, 54, 55, 59, 61, 102, 106].

#### 3.4.4 Migration and Location

##### 3.4.4.1 Migration

Only two studies investigated migration status in relation to mobile intervention uptake, and results were mixed. One found that non‐Saudi nationals living in Saudi Arabia had lower uptake rates [98]; the other, a survey conducted in Germany, found that participants with a native language other than German were more likely to use health apps [77].

##### 3.4.4.2 Location

Eleven studies examined potential differences in uptake in relation to location. Four studies reported statistically significant findings, but the direction of effects varied. One study reported rural residents to be more likely than urban residents to use mobile interventions [87], whereas three others reported the opposite effect [34, 35, 88]. Rha et al. [64] reported differences between countries: US residents were less likely to use wearable health devices than South Korean citizens. One study reported mixed results: Urban residents were more likely to use physical activity‐related apps, but there was no urban–rural divide for diet‐related apps, use of wearables, or smart scales [103]. Two studies [49, 97] broke down user rates by region within Saudi Arabia and the United States, respectively, but did not statistically test for differences. The remaining three studies did not report statistically significant differences [52, 56, 61].

#### 3.4.5 Sexual Orientation

Sexual orientation was only considered in one study, where it was unrelated to health app uptake [102].

#### 3.4.6 Socioeconomic Position

##### 3.4.6.1 Education

Thirty‐one studies investigated the relationship between education and mobile intervention uptake. Twenty‐one studies reported statistically significant differences; they consistently reported that a higher education level was associated with higher uptake rates [33, 34, 35, 36, 37, 38, 42, 45, 47, 52, 54, 55, 56, 59, 61, 88, 96, 98, 108, 109, 111]. Another two studies reported mixed findings. O'Loughlin et al. [89] found that higher educational attainment was only associated with physical activity app uptake but not with diet app uptake. Contrary results were reported by König et al. [79], who found educational attainment only to be positively associated with diet but not physical activity app use. One study reported a breakdown of education categories (high school, Bachelor's degree, and postgraduate) by app user category but did not statistically test for differences [97]. The remaining seven studies reported nonsignificant results [77, 81, 83, 84, 103, 106, 117].

##### 3.4.6.2 Income

Eighteen studies studied income in relation to mobile intervention uptake. Twelve reported statistically significant relationships between income and uptake, with higher income being consistently associated with higher uptake rates [35, 36, 37, 38, 39, 45, 47, 52, 54, 61, 96, 108]. Another three studies reported mixed findings. In one study, higher income was only associated with physical activity app uptake but not with diet app uptake [89]. In Shaw et al. [56], higher income was only significantly associated with uptake before controlling for other social inequality indicators. Finally, Rha et al. [64] suggest a moderation by location, with higher income only being associated with wearable device uptake in the United States but not in South Korea. One study did not statistically test for relationships but reported a breakdown of household income categories by app uptake (user, ex‐user, and nonuser [97]). Two further studies reported nonsignificant results [39, 59].

##### 3.4.6.3 Employment or Occupation

Employment or occupation was considered in 12 studies. Five of them reported statistically significant relationships. Holding a (full‐time) job was associated with increased uptake rates [47, 55, 96, 98]. Pontin et al. [86] reported inverse associations, with individuals in higher managerial positions being less likely to use health‐related apps. Another two studies reported mixed findings. In O'Loughlin et al. [89], being employed was only associated with using physical activity but not diet apps. In Zarnowski et al. [103], being employed was associated with using physical activity and diet apps and wearables but not with using smart scales. One study descriptively reported employment status by app user category without statistically testing for differences [97]. Four studies reported no significant results [34, 56, 77, 117].

##### 3.4.6.4 SEP Composite Score

Five studies used composite measures for SEP; four reported statistically significant relationships with mobile intervention uptake. Lower social grade, a low index of deprivation and lower self‐rated social class were associated with reduced likelihood of wearable uptake [87, 117], as was a lower SEP score [106]. In Hahn et al. [42], a lower SES score was only related to a reduced likelihood of mobile intervention app use in men but not in women.

#### 3.4.7 Relationship Factors

##### 3.4.7.1 Marital or Relationship Status

Potential associations between marital or relationship status and mobile intervention uptake were tested in 14 studies. Three of them reported statistically significant results. Being married was associated with an increased likelihood of using a mobile health intervention in two studies [56, 88] and with a lower likelihood in another [109]. In the same study, being widowed was associated with an increased likelihood of intervention uptake [109]. In Zarnowski et al. [103], only the use of physical and diet apps was more prevalent in singles, whereas there was no significant association with using wearables or smart scales. The remaining nine studies reported null results [33, 34, 36, 37, 39, 47, 64, 96, 98].

##### 3.4.7.2 Having Children

The relationship between mobile intervention uptake and having children was tested in five studies, of which two reported statistically significant results. In one of these studies, having children was associated with a lower likelihood of intervention uptake [38], whereas in the other, it was associated with an increased likelihood of intervention uptake [96]. Two more studies reported mixed findings. In Rha et al. [64], having children was only positively associated with wearable device uptake in the United States but not the South Korean subsample. In Zarnowski et al. [103], having children was associated with a lower likelihood of using physical activity and diet apps but not with using wearables or smart scales. One reported null results [98].

##### 3.4.7.3 Cohabitation

Living in one household with others was taken into consideration in two studies. Lee et al. [109] reported that individuals living with friends were more likely to use mobile health interventions than individuals living alone. In Zarnowski et al. [103], living with others was only positively associated with wearable use but not with the uptake of physical activity or diet apps or smart scales.

##### 3.4.7.4 Having Sick Family Members

Only one study took into account caring for sick relatives, which was positively associated with health app uptake [33].

#### 3.4.8 Health‐Related Social Inequality Indicators

##### 3.4.8.1 Having Chronic Conditions or Comorbidities

The relationship between having chronic conditions or comorbidities and mobile intervention uptake was tested in 12 studies. Six of them reported statistically significant findings. In five of them, individuals with chronic conditions were more likely to use health apps than individuals without chronic conditions [39, 52, 77, 98, 109]. In one study, chronic conditions were associated with a decreased likelihood of using wearables [61]. One additional study reported mixed results: The likelihood of using a physical activity app was reduced in individuals with a chronic condition, whereas it was unrelated to the use of diet apps, wearables, and smart scales [103]. The remaining five studies did not find significant associations [34, 36, 37, 45, 47].

##### 3.4.8.2 Health Insurance Coverage or Utilization

Six studies examined the relationship between health insurance coverage or utilization and uptake of mobile health interventions. Having health insurance was positively associated with mobile intervention uptake in two studies [34, 61]. One study descriptively reported the type of health insurance respondents had by app uptake [49]. The remaining three studies reported null results [47, 56, 59].

##### 3.4.8.3 Regular Provider or Primary Care Visits

The association between regular provider or primary care visits and mobile intervention uptake was tested in four studies. All reported nonsignificant findings [34, 36, 37, 39].

##### 3.4.8.4 Disability Status

One study took into account disability status and found that having a limiting disability reduced the likelihood of mobile intervention uptake [87].

### 3.5 Engagement

**FIGURE 3:**
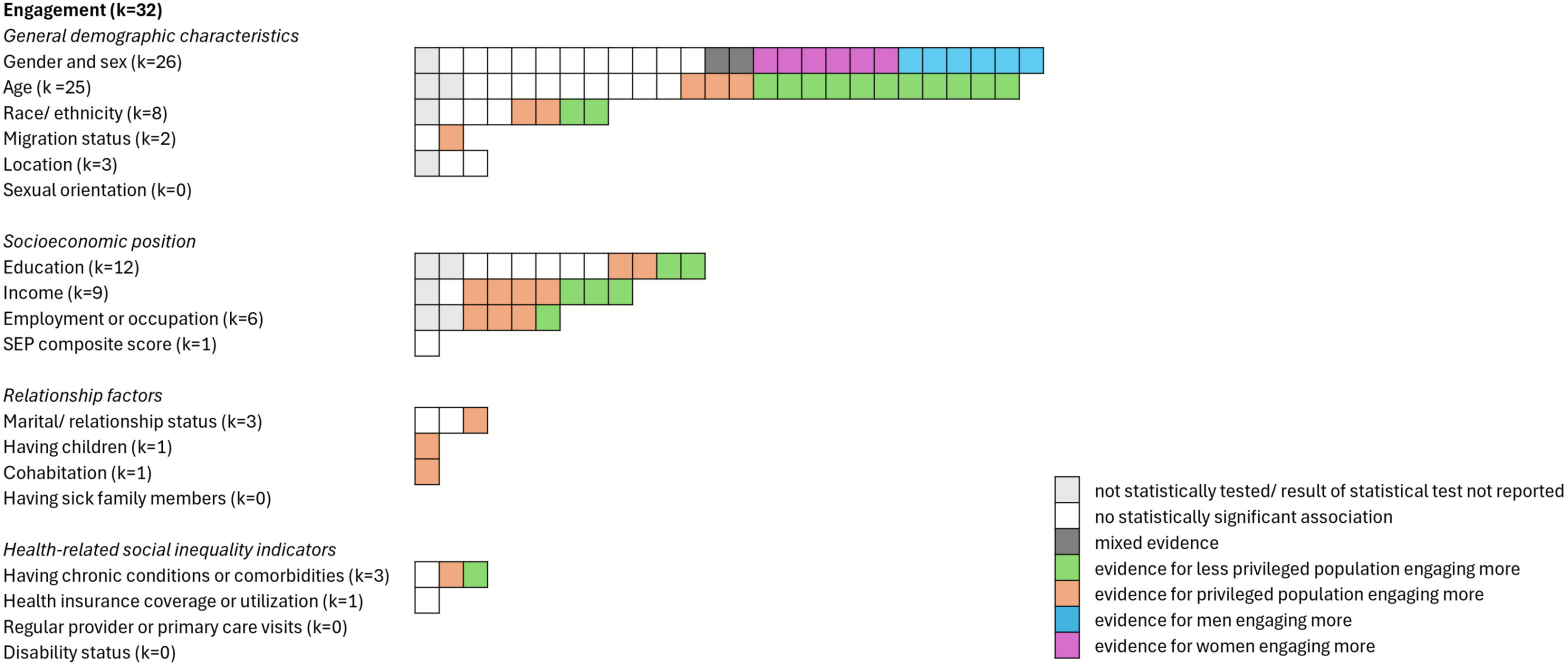
Visual summary of the included studies on engagement with the intervention (*k* = 32) by social inequality indicator. In each row, a box represents one study examining the relationship between the inequality indicator and engagement with the intervention. Privileged populations are those typically experiencing better access to healthcare and better health outcomes, such as White individuals, individuals with higher SEP as indicated by higher level of education or income or full‐time employment, individuals in a relationship but without care responsibilities, or individuals with access to health insurance and no chronic conditions. Results for genders are mixed in this regard (e.g., women reporting greater uptake of interventions but fewer benefits), and findings are thus reported separately for men and women.

See Figure 3 for a visual summary of the findings.

#### 3.5.1 Gender and Sex

Potential gender or sex disparities in engagement were tested in 26 studies. Twelve studies reported significant differences. Men engaged more in six studies [46, 51, 52, 65, 66, 92], whereas women engaged more than men in another six studies [50, 73, 86, 93, 100, 104]. Two studies reported mixed results. Torres et al. [75] reported higher retention in men but no sex differences for intervention component use. Molina et al. [76] found that women engaged more than men with some intervention components. One study reported gender differences descriptively without a statistical test [97]. Nine studies found no gender differences in intervention engagement [38, 40, 63, 78, 80, 83, 88, 90, 101, 114, 115].

#### 3.5.2 Age

Relationships between age and intervention engagement were tested in 25 studies. Fourteen reported significant results. Engagement was lower in younger users in 10 studies [38, 40, 46, 65, 73, 75, 86, 92, 100, 104]. In another three studies, engagement was higher in younger than older users [51, 52, 63]. Younger users were more likely to use social intervention components in one study [80]. Two reported results only descriptively [74, 97]. Nine studies reported nonsignificant associations [50, 70, 78, 83, 85, 88, 90, 93, 101].

#### 3.5.3 Race and Ethnicity

Potential disparities based on race and ethnicity were tested in eight studies. Four studies reported significant results. Black and African American users engaged more with mobile interventions in Lewis et al. [46]. Similarly, White individuals were more likely to abandon the intervention according to Nuss and Li [50]. Pevnick et al. [51], however, reported that early adopters of mobile health technology were more likely to be White, and Rising et al. [52] reported the engaged users were less likely to be Black. Gorny et al. [110] only reported a breakdown by ethnicity descriptively. Three studies reported null findings [38, 40, 75].

#### 3.5.4 Migration and Location

##### 3.5.4.1 Migration

Migration status was considered in two studies. Pevnick et al. [51] reported that early adopters of digital health technology were more likely to have English as their native language and thus no migration history. O'Loughlin et al. [90], however, found no differences based on migration.

##### 3.5.4.2 Location

Location was examined in three studies. There were no differences in engagement based on geographical location in Rising et al. [52] or region in Macridis et al. [88]. Alshathri et al. [97] only reported a descriptive breakdown by region within Saudi Arabia.

#### 3.5.5 Socioeconomic Position

##### 3.5.5.1 Education

Relationships between education and intervention engagement were tested in 12 studies. Four reported statistically significant results. In two studies, higher educational attainment was associated with increased engagement [78, 92], whereas in another two studies, higher educational attainment was associated with decreased engagement [50, 80]. Two studies only reported descriptive breakdowns [97, 110]. Six studies reported no significant associations [38, 40, 52, 83, 88, 101].

##### 3.5.5.2 Income

Nine studies examined relationships between income and intervention engagement. Seven studies reported statistically significant associations. One study reported intervention engagement to be higher in high‐ versus low‐income countries [67], and another study reported increased engagement in individuals with higher income [40]. One study reported that individuals with higher income were more likely to engage with diet tracking components [80], and another reported high‐income users to be especially engaged with physical activity‐related components [92]. However, three studies reported engagement to be lower in individuals with higher income [50, 51, 52]. Another study only reported a descriptive breakdown of categories [97]. One study reported null findings [38].

##### 3.5.5.3 Employment or Occupation

Six studies investigated employment or occupation in relation to intervention engagement. Four reported significant findings. Pevnick et al. [51] indicated that early adopters of health technology were more likely to be employed by a health insurance company. Stehr et al. [80] found stronger engagement in employed users. Friel et al. [40] reported engaged intervention users to be more frequently employed full‐time compared to former intervention users, whereas Fischer et al. [78] reported that intervention completers were more often retired and less often employed. Two more studies only provided descriptive results without statistical tests [86, 97].

##### 3.5.5.4 SEP Composite Score

SEP composite measures were related to engagement in one study that reported no significant associations [90].

#### 3.5.6 Relationship Factors

Having sick family members and sexual orientation were not studied with regard to intervention engagement.

##### 3.5.6.1 Marital or Relationship Status

Differences in engagement based on marital or relationship status were tested in three studies. Current intervention users were more likely to be in a relationship than former users in one study [101]. Two other studies reported no significant association [78, 88].

##### 3.5.6.2 Having Children

Coughlin et al. [38] reported that having children was associated with reduced intervention engagement.

##### 3.5.6.3 Cohabitation

Friel et al. [40] indicated that current intervention users were more likely to cohabit than former intervention users.

#### 3.5.7 Health‐Related Social Inequality Indicators

Regular provider or primary care visits and disability status were not studied in relation to mobile intervention engagement.

##### 3.5.7.1 Having Chronic Conditions or Comorbidities

The relationship between chronic conditions or comorbidities and mobile intervention engagement was addressed in three studies. Two of them reported statistically significant findings. Individuals with at least one chronic condition used intervention features such as challenges, rewards, and badges less frequently [46]. Rising et al. [52] reported users with chronic conditions engage more frequently with app‐based interventions. Pevnick et al. [51] reported no effects.

##### 3.5.7.2 Health Insurance Coverage or Utilization

One study tested the relationship between health insurance coverage or utilization and intervention engagement, reporting null findings [51].

### 3.6 Effectiveness

**FIGURE 4:**
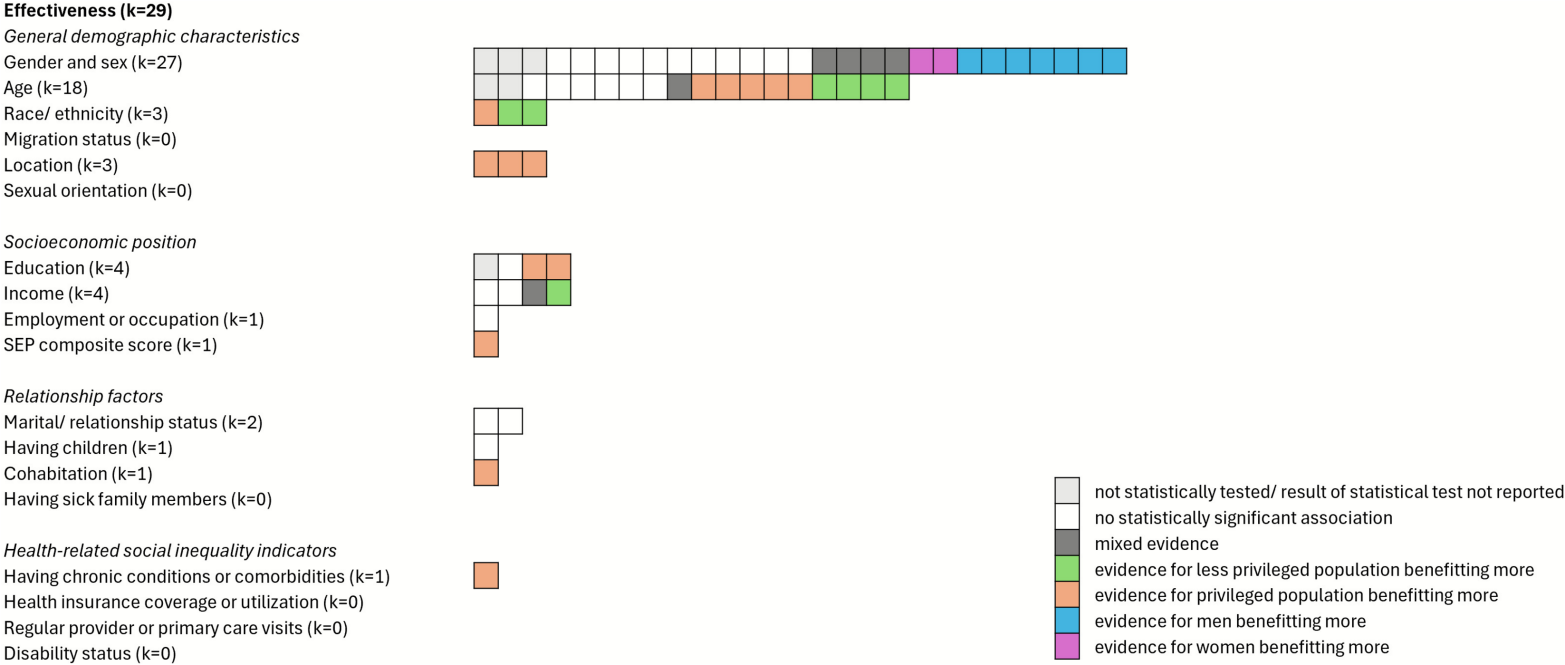
Visual summary of the included studies on intervention effectiveness (*k* = 29) by social inequality indicator. In each row, a box represents one study examining the relationship between the inequality indicator and intervention effectiveness. Privileged populations are those typically experiencing better access to healthcare and better health outcomes, such as White individuals, individuals with higher SEP as indicated by higher level of education or income or full‐time employment, individuals in a relationship but without care responsibilities, or individuals with access to health insurance and no chronic conditions. Results for genders are mixed in this regard (e.g., women reporting greater uptake of interventions but fewer benefits), and findings are thus reported separately for men and women.

A visual summary of the findings is presented in Figure 4.

#### 3.6.1 Gender and Sex

Twenty‐seven studies tested for potential gender‐ or sex‐related disparities. Twelve studies reported statistically significant differences. Men were more successful in losing weight than women [48, 68, 71, 105] and also reported higher levels of physical activity due to the intervention [86, 91]. Chin et al. [66] also reported men to lose more weight but also to experience greater weight regain after the intervention. In Lewis et al. [46], the self‐reported helpfulness of wearables including social features was higher in men than in women. Labonté et al. [63] furthermore suggest that gender may interact with engagement and weight status at baseline, with women benefiting more than men if their engagement is high. Jacobs et al. [69] reported greater changes in Body Mass Index (BMI) for women compared to men. In Hendrie et al. [100], women increased both the amount and types of vegetables consumed, whereas men only increased the types of vegetables consumed. Silberman et al. [57] reported that both men and women lost weight at equal rates, but these findings were qualified by an interaction with age, where women aged 18–39 lost weight at a higher rate than women aged 40–59. For men, there were no differences based on age group. One study reported mixed results: Although there were no gender differences in physical activity, men indicated more strongly that Pokémon Go was beneficial to their health [85]. Two studies only reported results descriptively without a statistical test [94, 95], and one study provided a statistical test, but no information about the direction of the significant effect of gender [67]. Eleven studies reported null results [41, 60, 62, 75, 76, 99, 112, 113, 114, 115, 116].

#### 3.6.2 Age

Eighteen studies tested for age effects in mobile intervention effectiveness. Ten studies reported statistically significant differences. Calorie intake was lower in older age groups [62], vegetable intake higher [100], and levels of physical activity higher [91], and older age groups were found to be more successful in losing weight compared to younger age groups [57]. Age groups also differed in the type of physical activity engaged in, with cycling and running being more popular in adults between 30 and 60, whereas going to the gym was more popular with younger adults. Generally, adults between 30 and 60 also moved less than other age groups [86]. Three studies found that older adults showed smaller reductions in BMI than younger adults [68, 69, 105] and reported higher body weight in older versus younger users [67]. Younger adults found wearables with social features more helpful compared to older adults [46]. Two studies only reported results descriptively without providing statistical tests [63, 94]. Six studies found no effects of age on effectiveness [32, 41, 48, 60, 75, 112].

#### 3.6.3 Race and Ethnicity

Three studies addressed potential disparities due to race and ethnicity. All of them reported statistically significant findings. White individuals were more successful in losing weight than Black or Hispanic individuals in one study [41] and less successful in another [75]. White participants found wearables with social features less helpful than participants of other ethnicities [46].

#### 3.6.4 Migration and Location

Migration status was not studied in relation to intervention effectiveness. The relationship with location was tested in three studies, all of which reported statistically significant results. Urban residents reported higher levels of physical activity compared to rural residents [91]. People living in postal code areas with higher median grocery store access also reported consuming more fruits and vegetables, less sugar‐sweetened beverages, and lower BMIs [31]. Jacobs et al. [69] reported on continent‐level differences with European users losing more weight than American users, who again lost more weight than African and Latin American users.

#### 3.6.5 Socioeconomic Position

##### 3.6.5.1 Education

Four studies investigated education, out of which two reported significant associations with intervention effectiveness. Education was positively associated with behavior change [60]. Similarly, in postal codes with above median education, individuals report higher fruit and vegetable consumption and less fast food and sugar‐sweetened beverage consumption [31]. Hamaya et al. [95] only provided descriptives but not a statistical test. Cheng et al. [112] reported no significant relationships between education and intervention effectiveness.

##### 3.6.5.2 Income

Four studies tested the effects of income, with one study reporting significant results. One more study reported mixed findings: In high‐income postal code areas, individuals consume more fruits and vegetables, less fast food, less sugar‐sweetened beverages, but also a higher BMI [31]. Two further studies reported null findings [67, 68].

##### 3.6.5.3 Employment or Occupation

May et al. [48] reported no significant relationships with employment.

##### 3.6.5.4 SEP Composite Score

Pontin et al. [86] indicated that individuals from more affluent areas were more likely to meet physical activity guidelines.

#### 3.6.6 Relationship Factors

Having sick family members and sexual orientation were not studied in relation to intervention effectiveness.

##### 3.6.6.1 Marital or Relationship Status

Two studies tested for potential differences in effectiveness based on marital or relationship status, but results were nonsignificant [48, 112].

##### 3.6.6.2 Having Children

One study tested the effects of having children; results were not statistically significant [48].

##### 3.6.6.3 Cohabitation

Living with one to two children in the household was positively associated with weight loss [71].

#### 3.6.7 Health‐Related Social Inequality Indicators

Health insurance coverage or utilization, regular provider or primary care visits, and disability status were not studied in relation to mobile intervention effectiveness. One study reported that participants with a chronic condition found social features in wearables more helpful than participants without a chronic condition [46].

## 4 Discussion

Mobile interventions can be delivered to many people at low cost and potentially even in remote locations. Yet evidence is accumulating that they might not benefit all equally, contributing to a digital health divide [120]. The results of the present systematic review of observational studies of mobile intervention uptake, engagement, and effectiveness at least partly support this claim. They point towards a divide in intervention uptake, based on socioeconomic factors such as education and income, and also provide some support for earlier claims that low SEP populations might benefit less from digital interventions [17]. Yet most other findings are either heterogeneous or based on only a small number of available studies, pointing towards important research gaps and indicating at least some uncertainty regarding the existence of a divide, at least for exclusively mobile interventions.

### 4.1 Inequalities Based on Gender and Sex

Most available studies addressed potential differences in exclusively mobile intervention uptake, engagement, and effectiveness based on general demographic characteristics such as gender, sex, and age. Half of the included studies on gender or sex found that women were more likely to use mobile interventions; most others found no gender differences. This is in line with several studies reporting greater interest in diet and health in women [121]. At the same time, it might also reflect the gender health gap, which indicates that women spend more years in bad health than men, partly because of potential gender biases in healthcare, where women's health issues are sometimes overlooked or misdiagnosed [122]. The potential unsatisfactory experiences with the healthcare system might then drive them to seek digital alternatives. Interestingly, sex and gender differences in uptake do not necessarily translate into differences in intervention engagement. In the present review, about half of the included studies reported gender or sex differences in engagement, but findings were heterogeneous as to which group showed more engagement. These findings may reflect both gender‐specific needs regarding intervention design [123, 124] as well as gender‐specific barriers to intervention engagement. Especially women in midlife frequently report increased stress levels due to having to balance work and care responsibilities, which leads many to neglect their own health [125]. Similarly, the majority of studies reporting significant gender or sex differences in effectiveness found that men benefited more than women. This is in line with findings from a recent umbrella review [23]. Indeed, men benefiting more from behavioral interventions for weight loss is a common finding, which might be due to sex differences in body composition changes [126, 127] but also to differences in caregiving responsibilities and time available for self‐care [128]. It can thus be concluded that, despite greater initial interest, women may not benefit from exclusively mobile interventions as much as they could. Better understanding the reasons behind gender differences in intervention uptake, engagement, and effectiveness can inform the development of more tailored interventions that better support all genders.

### 4.2 Inequalities Based on Age

A similar number of studies report an age gap in mobile intervention uptake; studies reporting statistically significant differences mostly found younger adults to be more likely to take up mobile interventions, likely due to their interest and familiarity with technology [129]. However, age differences could actually be due to generational rather than actual age effects, so it could be speculated that this gap may close with time. At the same time, age differences in mobile intervention uptake might also be rooted in stereotypes. Some healthcare professionals, who believe that technology‐based interventions might not be suited for older individuals, may not recommend or actively discourage digital interventions for older adults [130]. This gatekeeping could potentially further contribute to the divide [123]. Interestingly, in most studies reporting statistically significant age differences, older adults were found to engage more with exclusively mobile interventions than younger adults. This may indicate that older adults see more need to change their behavior to protect against health risks [131]. Evidence regarding effectiveness was again inconsistent, which may again support the notion that older adults not benefiting from digital interventions is rather a myth that requires debunking [23].

### 4.3 Inequalities Based on Race/Ethnicity, Migration, and Location

Findings regarding race and ethnicity as well as migration status were mixed. Only about half of the included studies reported statistically significant differences, and the conclusions were inconsistent on which ethnic group (White, Black, or adults of other ethnicities) most frequently used and engaged with mobile interventions. The lack of detailed information on intervention content and the inclusion of a broad range of mobile interventions make it difficult to identify specific factors contributing to the reduced uptake and engagement in certain ethnic groups. Previous research highlighted the importance of cultural tailoring [132]. It also stresses that ethnic disparities may be interrelated with other inequality indicators such as income or education. However, due to the variation in the number and type of social inequality indicators taken into account in the included studies, we were unable to take these potential interdependencies into account.

Potential inequalities based on location were studied at two levels. First, some studies focused on within‐country differences, that is, differences between rural and urban users. Results were inconclusive. Although digital health technologies, including mobile interventions, are seen as a lever to improve health promotion in rural areas with limited access to health care [133], the studies included in this review suggest that mobile interventions may be less effective in rural areas. It is important to note that rural areas often do not only lack healthcare providers but also opportunities for procuring healthy foods or spontaneous physical activity, as the public transport network is often less dense [134, 135]. Without environmental changes through policy or other interventions to support these factors [136], the effectiveness of digital interventions will naturally be limited. Second, a small number of studies reported on between‐country differences, with inconsistent results: Whereas uptake of wearables was higher among South Korea versus US adults [64], intervention users from Europe and the United States, that is, the Global North, seemed to benefit more than intervention users from Africa and Latin America, that is, the Global South [69]. More research is urgently needed to shed light on potential between‐country disparities [120]; this is especially urgent because commercial apps and wearables are typically available globally.

### 4.4 Inequalities Based on SEP

Compared to most other inequality indicators addressed in the present review, the evidence for a divide based on SEP was the most consistent. For uptake, education and income seem to be most important. Indeed, although access to broadband internet and smartphones seems to be ubiquitous, especially in industrialized countries, the divide in access to digital technologies persists [137], even in countries such as the United States or Germany [138, 139], extending to mobile intervention use. Similarly, knowledge about the relationships between physical activity and dietary behaviors and health and the opportunities that mobile technology may provide for health promotion are likely correlated with education [140]. Income was also related to mobile intervention engagement, although the results were mixed as to whether high‐ or low‐income groups were more engaged. The results for intervention effectiveness were also inconsistent, making it unclear whether meaningful engagement and resulting intervention effectiveness can be achieved in low SEP populations once access barriers are addressed. A prior systematic review on digital interventions for physical activity suggests that this may not be the case, as it found that these interventions were only effective in high but not low SEP populations [17]. Although an increasing number of mHealth interventions are codeveloped with end‐users, researchers often struggle with recruiting diverse populations [141], and the resulting interventions therefore might not cater to the specific needs of low SEP populations.

### 4.5 Relationship and Health‐Related Factors

Many social inequality indicators, including sexual orientation, household composition and resulting care responsibilities, or health insurance coverage, were rarely studied. As a result, drawing conclusions regarding their potential influence on mobile intervention uptake, engagement, and effectiveness is difficult. Future (digital) intervention research urgently needs to consider these factors to close this research gap and determine whether these indicators require further attention in the development of (exclusively) mobile interventions.

### 4.6 Strengths and Limitations

The present review summarizes a total of 88 studies from 87 publications that were identified through an extensive search that was supported by a research librarian. It thus provides a comprehensive overview of the current state of knowledge regarding potential social inequalities in the uptake, engagement, and effectiveness of exclusively mobile interventions, which continue to grow in popularity [142]. Complementing prior research (e.g., Szinay et al. [20] and Western et al. [17]), which often focused on randomized‐controlled trials that may also suffer from unequal participation [24, 26], this review specifically focused on observational designs such as surveys, which often used large representative samples or users of commercially available mobile interventions with a much broader reach. However, the focus on observational studies rather than randomized‐controlled trials (cf. König et al. [23] and Szinay et al. [20]) limits the conclusions regarding intervention effectiveness, which were often based on group comparisons at a single time point or, in rare cases, assessed subjectively. Although a broad range of social inequality indicators were considered, conclusions about many remain limited due to a small number of available studies. Moreover, excluding studies that exclusively focused on individuals with chronic conditions was deemed necessary due to the large body of literature and to ensure comparability of study findings. Yet this may have led to certain aspects of the PROGRESS‐Plus framework, such as disability, being underrepresented in this review. At the same time, many included studies used general population samples that did not exclude participants for having medical conditions; thus, one study [87] was included that explicitly tested for effects of having a disability.

It is furthermore important to note that although the quality of most of the included studies was good or fair, we had to adjust the use of the NIH tool [30] so that it fits the purpose of the present review. We are convinced that moving from a rating of *good*, *fair*, or *poor* for each question to answering each question with *yes* or *no* and splitting some of the questions into subquestions (i.e., 4a,b; 5a,b; 9a–c; and 11a–c) has increased objectivity and preciseness of the risk of bias assessment. However, there are a few questions that appeared not relevant when the observational study assessed was of cross‐sectional nature (i.e., Questions 6, 7, and 10), when outcomes were assessed at the same time as exposure with no external and/or formal assessor involved (Question 12) or when secondary data analyses were conducted using complete datasets (Question 13). In these cases, we scored the question as either no or not applicable but still labeled the question as green because it seemed unfair to label it as red, although these were valid aspects associated with the chosen study design. As such, the overall conclusion that most studies were of good or fair quality might need to be interpreted with some caution.

### 4.7 Implications for Practice and Future Research

The review found that mobile intervention uptake differs by SEP, which has important implications for practitioners using or considering mobile interventions for health promotion. It points to a need to thoroughly precheck the chosen mobile intervention for factors such as ease of use and accessibility. Collaborating closely with members of the target group in reviewing different available mobile interventions for their usability, acceptability, and appropriateness can help select the most suitable option for uptake. However, it cannot be assumed that simply providing a mobile intervention to users will lead to uptake. Additional steps might be required, such as providing supplementary (in‐person) support during the initial stages of setting up and familiarization with the mobile intervention to enable effective long‐term use and address privacy and technical concerns.

Although the present review exclusively focused on mobile interventions without any in‐person support, others already raised the question as to whether, especially for underserved populations, in‐person components may be crucial for promoting uptake, engagement, and effectiveness [20]. So far, the literature has produced inconclusive results regarding whether combining digital and nondigital intervention components is beneficial. For instance, an early meta‐analysis indicated that adding a digital component to standard care improved weight loss outcomes [143], whereas another later meta‐analysis was unable to replicate this finding [144]. Also when comparing digital and nondigital interventions in the context of cardiovascular risk reduction, a recent meta‐analysis did not find statistically significant differences [145]. Still, other work suggests that combining multiple modes of delivery, including personal contacts, may increase intervention effectiveness [146, 147]. Importantly, the literature is heterogeneous and unsystematic, given the various forms of digital and nondigital intervention delivery that can be applied in practice [148], and research is especially sparse regarding who benefits most from which delivery mode or combination of delivery modes [143]; this is an important gap that needs addressing in future studies.

Previous research has highlighted that a variety of factors ranging from the individual up to macrofactors affect uptake [137], with lower educational status and lower income being associated with lower digital skills and competencies [149]. To support these populations, efforts to improve digital health literacy and skills are essential and might benefit individuals beyond mobile health interventions.

Interestingly, many findings showed statistical significance around the 5% mark, highlighting the contrasting findings across uptake, engagement, and effectiveness. Given these contrasting findings and the potential for widening existing health inequalities, future research is urgently required. It is important that future studies are adequately powered to detect potential subgroup differences and account for multiple comparisons in their analyses.

## 5 Conclusions

Although a wide range of factors could potentially influence the uptake, engagement, and effectiveness of exclusively mobile health interventions for weight‐related behaviors [21], the focus of most studies lies on socioeconomic disparities rooted in gender/sex, age, SEP, and, to a lesser extent, race and ethnicity. By focusing on observational studies and including many large‐scale surveys and retrospective analyses of available data from commercial apps, the present review identified several factors potentially linked to mobile intervention uptake. We found that available mobile interventions are predominantly used by younger and more affluent adults, leaving populations at risk for weight‐associated conditions such as older adults and low‐SEP populations behind [150, 151, 152, 153]. Results for engagement and effectiveness were mixed, suggesting that reducing barriers to intervention uptake might be most promising for reducing the digital health divide. Thus, a stronger focus on intervention uptake in other study designs, including RCTs, and systematic reviews (cf. [23]) and a broader range of social inequality indicators addressed are required to gain a better understanding of who even considers using these interventions and to subsequently design interventions specifically for disadvantaged populations to improve health for all.

## Author Contributions

**Laura M. König:** conceptualization, data curation, formal analysis, funding acquisition, investigation, methodology, project administration, visualization, writing – original draft. **Cynthia C. Forbes:** conceptualization, funding acquisition, investigation, methodology, writing – review and editing. **Heide Busse:** conceptualization, funding acquisition, investigation, methodology, writing – review and editing. **Ann DeSmet:** conceptualization, formal analysis, funding acquisition, investigation, methodology, visualization, writing – review and editing. **Dorothy Szinay:** conceptualization, formal analysis, funding acquisition, investigation, methodology, writing – review and editing. **Jin Wan:** investigation, writing – review and editing. **Zhirui Guo:** investigation, writing – review and editing. **Eline S. Smit:** conceptualization, formal analysis, funding acquisition, investigation, methodology, project administration, supervision, writing – original draft.

## Funding

This work was partially funded through a networking grant provided by the European Health Psychology Society (granted to H.B., A.D., C.C.F., L.M.K., E.S.S., and D.S.).

## Conflicts of Interest

The authors declare no conflicts of interest.

## Supporting information


**Data S1:**Supporting Information

## Data Availability

The datasets generated and/or analyzed during the current study are available from the Open Science Framework (https://osf.io/qs5vy/).
